# An overview of HPAI H5N1 clade 2.3.4.4b and its emerging threat in mainland Australia: Identified knowledge gaps

**DOI:** 10.1016/j.onehlt.2025.101292

**Published:** 2025-12-10

**Authors:** Pan Zhang, C Raina MacIntyre

**Affiliations:** Biosecurity Program, Kirby Institute, Faculty of Medicine and Health, University of New South Wales (UNSW), Sydney, Australia

**Keywords:** H5N1, Clade 2.3.4.4b, Avian influenza, HPAI, Incursion risk, Australia, Antarctica, Heard Island

## Abstract

The highly pathogenic avian influenza (HPAI) H5N1 virus, particularly clade 2.3.4.4b, is currently causing a panzootic. The threat of its incursion into mainland Australia is escalating, especially following its detection in Antarctica and recently on Australia's sub-Antarctic territory, Heard Island. Comprehensive research on this emerging risk remains limited, partly due to ongoing rapid genomic mutations and evolving epidemiological dynamics. This review provides an in-depth analysis of clade 2.3.4.4b H5N1 and highlights critical knowledge gaps that must be addressed to mitigate the potential threat to mainland Australia.

## 1 Introduction

### 1.1 Avian influenza and current panzootic situation of clade 2.3.4.4b HPAI H5N1

Avian influenza, caused by the influenza A virus, is a highly contagious viral disease that primarily infects domestic and wild birds [1]. Highly virulent strains can cross species barriers by binding to specific sialic acid receptors expressed in mammalian hosts [2]. The complex nature of avian influenza viruses (AIVs) and long distances of wild bird migration facilitate their worldwide spread; however, the prevalence of specific subtypes varies geographically and is associated with various ecological and epidemiological factors [3].

Over the past six decades, the highly pathogenic avian influenza (HPAI) H5N1 virus has undergone extensive evolutionary changes, initially causing sporadic outbreaks among poultry in Asia and occasional cross-species transmission to humans. It subsequently diversified into multiple clades, including 2.2, 2.2.1.2, 2.2.2, 2.3.2.1, and 2.3.4.4, leading to two major surges before 2020; however, its geographic distribution remained largely confined to Asia [4]. Since 2020, the clade 2.3.4.4b HPAI H5N1 was initially detected in both wild and domestic birds within Europe and subsequently became dominant across multiple continents by late 2021. Beyond avian hosts, the virus has also infected various mammalian species, highlighting the vigilance regarding spill-over transmissions between species [3]. Since 2022, H5N1 clade 2.3.4.4b has spread aggressively among wildlife in South America [5] and also reached Antarctica in October 2023 [6]. Australia had remained the sole continent unaffected by this clade until recently. In November 2025, Australian scientists reported unusual mortality events among elephant seals during an expedition to Heard Island, an Australian sub-Antarctic territory. Subsequent laboratory analyses confirmed the presence of the panzootic clade 2.3.4.4b HPAI H5N1. This detection marks its first incursion into the Australian territory and highlights the imminent risk of its spread to mainland Australia [7].

### 1.2 The current situation of escalating HPAI H7 poultry outbreaks in Australia

Since 2020, Australia has experienced a significant increase in poultry outbreaks caused by strains distinct from the globally circulating clade 2.3.4.4b H5N1, with a total of 20 HPAI outbreaks reported. Notably, 16 of these outbreaks occurred in 2024 alone. In contrast, only seven HPAI outbreaks had been recorded between 1976 and 2020 [8]. These recent outbreaks included highly pathogenic H7 subtypes detected across multiple states. The escalation in poultry outbreaks has led to substantial losses, with approximately 1.8 million bird fatalities reported in 2024 [9].

Australia has historically benefited from its geographic isolation as an island continent, which has likely contributed to the low incidence of avian influenza epidemics. Additionally, robust biosecurity measures, supported by legislation, have continued to protect the nation from exotic pests and emerging disease threats, despite increasing population movement and trade volumes [10]. Furthermore, natural biogeographical barriers, such as Wallace's Line, limit the migration of certain wild bird species from Asia to Australia, further reducing the risk of disease introduction [11]. However, the recent transmission of clade 2.3.4.4b HPAI H5N1 to Antarctica and Heard Island, along with the escalating situation of HPAI H7 poultry outbreaks within Australia, heightens concerns about the potential introduction and establishment of the virus in the mainland. In particular, certain wild seabirds may have the capacity to undertake long distances while infected, and their migratory flyways may extend from sub-Antarctic regions to mainland Australia [12].

## 2 Key genomic changes in clade 2.3.4.4b HPAI H5N1 virus

### 2.1 Unique NA features enhance pandemic potential

Since its emergence in 1996 among geese in Guangdong, China (Gs/Gd lineage), the HPAI H5N1 virus has demonstrated a remarkable capacity for vast spatial spread across multiple countries, regions, and continents. This extensive inter- and intracontinental transmissibility likely results from the transition of the initially stable neuraminidase (NA) protein of the N1 subtype, mostly derived from Gs/Gd-like viruses, to the emergence of novel NA subtypes [13]. A recent study identified a long NA stalk domain in multiple isolates of clade 2.3.4.4b H5N1, including those obtained from dairy cattle [14]. This shift may have contributed to the significant rise in infections among mammalian species observed since 2020, affecting both domestic mammals and wild marine mammals [15]. Such characteristics in clade 2.3.4.4b H5N1 may also enhance the risk of human-to-human transmission, raising concerns about its potential to cause a pandemic [14].

### 2.2 Potential dual receptor-binding specificity of bovine HPAI H5N1 clade 2.3.4.4b to both α2,3- and α2,6-linked sialic acids

The adaptation of HPAI H5N1 clade 2.3.4.4b to mammalian hosts is unprecedented, as evidenced by the outbreak among dairy cattle in the United States in 2024. Before that, HPAI viruses had not been reported to infect cattle [16]. As of September 14, 2025, a total of 1079 cattle outbreaks have been documented across 17 states in the U.S. [17]. The spread of H5N1 in cattle highlights the virus's remarkable capacity for rapid genetic mutation and functional adaptation of key viral proteins, particularly those mediating host specificity. Cattle demonstrate a distinct distribution of epithelial cells in the alveoli of the mammary gland, which express receptors with affinity for the H5N1 virus. An initial study showed a unique dual banding-receptor preference for both α2,3- and α2,6-linkage sialic acids of bovine H5N1 clade 2.3.4.4b viruses [18]. Although subsequent studies have doubted this finding [19,20], more recent research indicates a slight binding preference of bovineH5N1 viruses for human-like α2–6-linked receptors [21]. Nevertheless, H5N1 viruses isolated from cattle retain a strong affinity to avian-like α2–3-linked sialic acid receptors [21]. The potential for dual receptor-binding specificity in bovine H5N1 viruses poses a significant threat of facilitating transmission in mammals.

### 2.3 Presence of mammalian-adapted PB2 mutations E627K & D701N in HPAI H5N1 clade 2.3.4.4b

Clade 2.3.4.4b H5N1 has exhibited key polymerase mutations, including E627K and D701N in the PB2 protein, which represent another marker of mammalian adaptation, highlighting the risk of zoonotic transmission between mammals and pandemic potential [22]. During the ongoing H5N1 panzootic, viral isolates from multiple countries, particularly those where mammal infections have been reported, have shown the E627K substitution in PB2 [23,24]. H5N1 outbreaks in Chile have primarily involved viruses carrying the D701N mutation in the PB2 protein [24]. The mutation has been detected in sea lions, a human case, and a sanderling. The D701N mutation has not been reported in association with onward transmission, although Chile's H5N1 outbreaks were likely genetically close to those reported in Peru. The sanderling and human cases both shared mutations, apart from D701N, with marine mammals in Chile [24]. Contrasting the traditional pathway where swine typically serve as intermediate hosts before the H5N1 virus adapts to humans [25], the potential spillover from marine mammals to humans underscores an emerging transmission route for H5N1 to humans.

## 3 Pathogenesis of clade 2.3.4.4b HPAI H5N1 in selected key animal models

### 3.1 Poultry

#### 3.1.1 Chickens

Compared to previously circulating H5 lineages, the dominant H5N1 clade 2.3.4.4b exhibits increased infectiousness and mortality among chickens [[26], [27], [28]]. Infected chickens exhibited higher viral shedding in oral secretions compared to faecal excretions [26]. A distinctive characteristic of HPAI H5N1 clade 2.3.4.4b in chickens is its relatively inefficient transmissibility within poultry populations compared to other species [29,30].

#### 3.1.2 Ducks

Waterfowl are the primary reservoir for AIV, facilitating genetic reassortment and continuous evolution [31]. Compared to chickens, HPAI H5N1 clade 2.3.4.4b has demonstrated higher infectiousness in ducks. Additionally, viral concentrations detected in water samples were higher than those observed in air and dust samples [29]. This suggests that shared water sources are likely the primary transmission pathway for H5N1 between wild waterfowl and domestic ducks. Furthermore, ducks exhibited an extended period of viral shedding compared to chickens [26,32], which may potentially enhance viral transmission within poultry populations. Feathers may also serve as an additional route of infection [33]. HPAI viruses, especially those of the Gs/Gd lineage, exhibit viral tropism in feathers among waterfowl. Waterfowl engage in a regular preening routine, which may enable H5N1 to enter the body through feather contact [34].

### 3.2 Mammals

#### 3.2.1 Domestic mammals: dairy cattle

The precise pathway through which the H5N1 virus enters cattle remains uncertain. Compared to respiratory secretions or droppings, milk from infected cattle has been found to exhibit higher levels of vial shedding [35,36]. Several studies indicate a viral tropism of mammary tissue in cows [[36], [37], [38]]. As a result, possible pathways include respiratory or indirect contact exposure with contaminated feed, water, or droppings at low levels of viral loads, while replicating primarily at the mammary site [39]. A direct infection of the mammary tissue through the mouth-to-teat canal transmission during the sucking procedure is also possible [40].

#### 3.2.2 Marine mammals: sea lions

The most probable transmission pathway for H5N1 outbreaks among marine mammals, such as seals and sea lions, is the oral route via a shared food source with wild birds [41]. Wild sea birds carrying HPAI H5N1 may have preyed on fish, including anchovies and sardines, which also constitute a food source for sea lions [42]. Environmental exposure is another potential mechanism of infection in marine mammals. Direct transmission has also been hypothesised, wherein marine mammals, including sea lions, may have consumed infected wild bird carcasses [43]. Although seabirds are not a primary diet for marine mammals, certain seabirds exhibit colony breeding behaviour in close association with marine mammalian populations. The shared habitats facilitate frequent interactions, increasing the likelihood of opportunistic predation of seabirds by marine mammals [44].

### 3.3 Humans

Human cases of HPAI H5N1 remain rare compared to infections in other mammalian species [45]. Most reported human infections have resulted from close contact with or the consumption of contaminated poultry or cattle. Historically, this transmission pathway has led to severe clinical conditions, including respiratory failure and encephalitis, and occasionally high fatality rates in young children [45]. While the ocular route has also been hypothesised, an experimental study found that the clade 2.3.4.4b HPAI H5N1 viruses do not exhibit a tissue tropism for human ocular cells [46]. The virus cannot currently replicate in mammalian ocular tissue [46], suggesting a low likelihood of transmission via the ocular route. Nonetheless, experiments on transmission mechanisms using a ferret model demonstrated the virus's ability to maintain virulence via ocular exposure [47]. Since ferrets and humans share similar respiratory physiology, with both species expressing sialic acids in their respiratory tract that display comparable influenza virus binding patterns [47], the risk of H5N1 infection through ocular exposure in humans remains a consideration.

## 4 Epidemiological risk factors influencing H5N1 spread

### 4.1 Wild bird migration

Many wild birds, especially waterfowl, migrate over long distances and have historically harboured AIVs [31]. Their movements predominantly follow north-to-south routes, characterised by seasonality that aligns with breeding and wintering routines. As a result, migratory flyways form, serving as essential drivers of the continuous circulation and vast geographic expansion of AIVs [48]. Globally, nine major migratory flyways have been identified, some of which -particularly in North America- overlap in certain island areas while remaining distinct along coastal regions. These overlaps could facilitate multiple introductory events of AIV in new locations [49].

The migratory patterns of wild birds along migratory flyways are primarily associated with food availability, breeding requirements, and weather conditions [50]. Changes in flyway landscapes may have altered migration patterns, thereby influencing the risk of avian influenza transmission [51]. In recent years, China has experienced a significant decline in rice paddy fields, resulting in a substantial decrease in stopover habitats along the East Asian-Australasian Flyway (EAAF), such as those in the Yangzi River Basin [52]. In response, migrating birds are increasingly congregating in the remaining rice paddy fields, thereby intensifying transmission dynamics within wild bird populations and between wild and domestic avian species. These congregation sites have thus become key environments for genomic reassortment events, facilitating the emergence of novel viral variants [52].

### 4.2 Live bird markets and poultry trade

Live poultry markets (LBM) are considered primary drivers in the spread of H5N1 outbreaks in Asia [53]. These markets serve as central hubs within poultry trade networks, where diverse bird species are collected and sold. Consequently, birds harbouring low pathogenic avian influenza (LPAI) viruses function as ideal reservoirs, potentially contributing to the emergence of new circulating variants [53]. Human exposure to AIVs through direct contact with infected birds or contaminated environments in these live bird markets is also considered high risk [54].

Globally, the poultry trade has been observed as a significant factor in the dissemination of HPAI H5N1 from Asia to Europe and Africa, likely occurring before the major migratory movements of wild birds [55]. In particular, China and India have been leading exporters of poultry to Europe [55]. While wild bird migration has played a major role in its intercontinental spread, the global poultry trade may have also contributed to the emergence and expansion of this dominant clade 2.3.4.4b H5N1 [55]. This underscores the multifaceted nature of H5N1 transmission, warranting continued surveillance of both avian migration and commercial poultry networks.

### 4.3 Farms

Farms have been identified as critical sites for mammalian outbreaks of HPAI H5N1 clade 2.3.4.4b in Europe and the Americas [56]. After initial introduction by wild birds, farms, like live bird markets, have become conducive environments for the sustained circulation of avian influenza and the emergence of novel variants. Several factors may facilitate these transmission dynamics, including contaminated bird droppings, shared food and water sources among domestic animals, and the use of contaminated equipment during milking procedures [25]. In particular, farms housing multiple animal species or located adjacent to wildlife habitats are vulnerable to cross-species exposures, thereby increasing the risk of spill-over and spill-back events. In addition to ongoing viral adaptations, the interstate movement and trade of domestic mammals or contaminated milk products likely contribute to farm-to-farm spread [35]. Cattle have increasingly been recognised as emerging “mixing vessels” specifically observed for clade 2.3.4.4b H5N1, with documented cross-species transmissions to various hosts, including cats, rodents, dogs, and poultry [38]. Remarkably, infected cattle have also been implicated in spill-back events to waterfowl, potentially contributing to further viral transmission [57].

### 4.4 Ecological factors: climate change

#### 4.4.1 Persistence of clade 2.3.4.4b HPAI H5N1 during warmer seasons

Although elevated temperatures in origin and destination regions have generally been associated with a decline in HPAI H5 transmission, the clade 2.3.4.4b H5N1 has exhibited unusual persistence in wild bird populations, particularly during 2022 [58]. The shift may have contributed to a subsequent spike in outbreaks during the summer months. While this trend suggests a potential increase in viral sustainability under higher temperatures, one possible hypothesis is that summer coincides with the primary breeding season for many migratory birds [59]. Newly hatched individuals are often immunologically naive, making them more susceptible to AIV infection [59]. Nevertheless, the persistence of HPAI H5N1 clade 2.3.4.4b during summer months indicates a shift in transmission patterns, potentially influenced by migratory dynamics and breeding cycles. These changes could have significant implications for viral spread and ecological interactions.

## 5 Avian influenza defense and migration: risks for Australia

### 5.1 Wallace's line

Compared to other regions, Australia has experienced relatively few avian influenza outbreaks [11]. Despite its geographical isolation, Wallace's line has been recognised as a crucial biological barrier, shaping the movement of migratory species and potentially influencing the incursion of AIV into Australia. Wallace's line is an invisible boundary that traverses Indonesia, separating Asiatic fauna from the Australian biogeographical realm [11].

Unlike the Northern Hemisphere, wild waterfowl in Australia typically do not undertake long-distance migration. Their movements are largely confined to regions southeast of the Wallace's line [11]. However, non-waterfowl species, such as shorebirds and seabirds, regularly migrate across extensive distances, linking Australia to Northern Hemisphere flyway systems [60]. These migrating species include members of families *Scolopacidae* and *Charadriidae*, as well as Short-tailed Shearwater. It is important to note that only a limited number of individuals from Eurasian populations reach the Austral-Papuan region, with even fewer regular land in Australia [11].

Wallace's line has been considered a natural defense against past incursions of HPAI H5N1 during two major surges of the virus. The panzootic clade 2.3.4.4b HPAI H5N1 has failed to establish in Australia over the past two years, as demonstrated by a study investigating migratory shorebirds and seabirds arriving in the country [61]. Although the faunal separation imposed by Wallace's line is not absolute, this invisible boundary may have contributed to limiting the incursion of the panzootic clade 2.3.4.4b HPAI H5N1 in Australia [62]. Nevertheless, as the virus continues to evolve in terms of virulence and adaptability, its impact on host species may influence ecological dynamics, including the movement patterns of migratory shorebirds and seabirds. Infection may impair the ability of these birds to complete long-distance migration, potentially altering established routes and increasing the viral spread to new regions. In this context, long-distance migration from Antarctica may present a novel pathway for H5N1 incursion in mainland Australia, particularly if infected birds can travel across continents while shedding the virus [6].

### 5.2 Migratory flyways between Asia and Australia

**Fig. 1:**
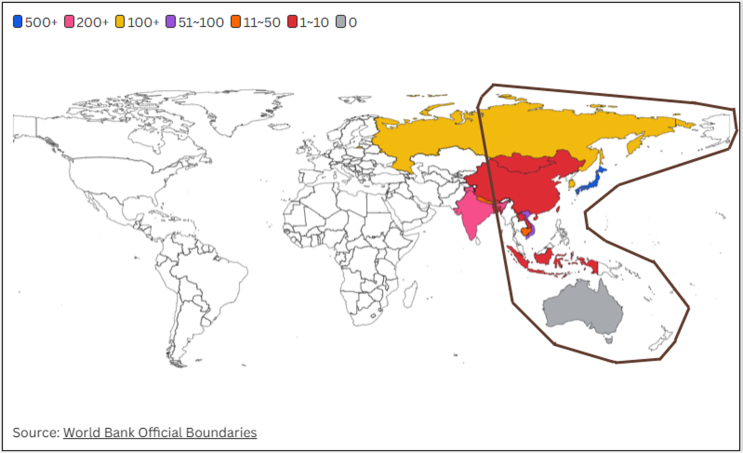
Reported number of highly pathogenic avian influenza (HPAI) H5N1 outbreaks in avian populations along the East Asian-Australasian Flyway (EAAF), 2021–2025. Outbreak data were obtained from the World Organisation for Animal Health [64]. The dark brown circle represents the East Asian-Australasian Flyway (EAAF), manually drawn by the author to illustrate the potential risk of HPAI H5N1 incursion into mainland Australia via migratory birds from this flyway, based on the map created by the East Asian-Australasian Flyway Partnership (EAAFP) [62]. (For interpretation of the references to colour in this figure legend, the reader is referred to the web version of this article.)

Each year, migratory shorebirds and seabirds arrive in Australia utilizing the East Asian-Australasian Flyway (EAAF) [62]. Their routes often originate from regions where HPAI outbreaks have been recorded, potentially exposing them to the virus before reaching Australia's eastern coastlines [63]. Notably, the commercial poultry industry is concentrated in Southeastern Australia, where proximity to migratory pathways may pose a risk for HPAI transmission. In recent years, the migration routes of some shorebirds along the EAAF have shifted, moving away from Australia's eastern coastline and instead traversing inland, migrating north to south across the continent. Although only a few HPAI outbreaks have been reported along the EAAF, and shorebird species known to carry the virus have not been documented migrating to Australia [63], the evolving migration patterns of arriving migrants continue to present a potential risk for disease introduction (Fig. 1).

Beyond long-distance migrants, many waterbirds, including egrets, pelicans, and ducks, primarily migrate between New Guinea and northern Australia, rather than following extended routes along the EAAF [11]. The Trans-Fly region of New Guinea may serve as a risk area for potential HPAI introduction into Australia, particularly given its proximity to West Papua, which reported the most recent H5N1 outbreak in 2006 [11]. Waterbirds in this region originate from diverse locations, including Asia, New Guinea, and Australia, further increasing the risk of avian influenza transmission.

### 5.3 Migratory flyways between Antarctica and Australia

On October 8, 2023, the presence of clade 2.3.4.4b H5N1 was detected in brown skuas on Bird Island, South Georgia. Subsequent testing confirmed additional positive cases in multiple avian and pinniped species [12]. Additionally, the Falkland Islands reported H5N1 infections in southern fulmars and albatrosses, likely resulting from wild bird migration originating from South America. In February 2024, the Argentine research station on the Antarctic Peninsula detected the virus in two skuas, marking the first confirmed cases of H5N1 in continental Antarctica [12]. In November 2025, an Australian sub-Antarctic territory, Heard Island, confirmed the detection of HPAI H5N1 clade 2.3.4.4b in elephant seals [7]. These events signify a remarkable expansion of clade 2.3.4.4b H5N1 into Antarctica and the Subantarctic regions.

**Fig. 2:**
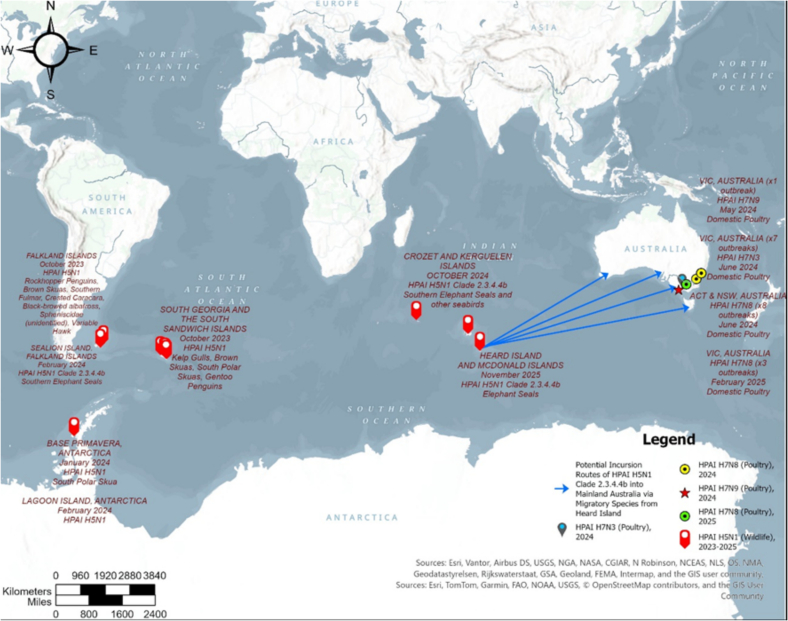
Reported detections of highly pathogenic avian influenza (HPAI) H5N1 in marine wild species in the Southern Ocean, and HPAI H7 outbreaks in domestic poultry in Australia, 2023–2025. The blue lines indicate the potential risk of HPAI H5N1 incursion into mainland Australia via migratory species from the Southern Ocean flyways. Outbreak data were obtained from the World Organisation for Animal Health [64], Clessin et al. (2025) [65], and Tracy (2025) [7]. Abbreviations: VIC, Victoria; ACT, Australian Capital Territory; NSW, New South Wales. (For interpretation of the references to colour in this figure legend, the reader is referred to the web version of this article.)

The emergence of clade 2.3.4.4b HPAI H5N1 in Antarctica and the surrounding subcontinent regions has raised significant concerns regarding its potential further expansion, particularly reaching mainland Australia via migratory species. Some seabird species, including brown skuas, exhibit circumpolar distributions and undertake extensive long-distance migrations [65]. Additionally, skuas have demonstrated susceptibility to H5N1, increasing concerns regarding the potential transmission of the virus from Subantarctic islands [66]. Several Subantarctic islands, situated between the Antarctic regions and Oceania, including Australia and New Zealand, serve as habitats for diverse species. Some seabirds, such as short-tailed shearwaters, typically breed in Australia while utilizing the polar front as a key forage area [67]. Despite the significant distance between the Subantarctic islands and Oceania, certain species, such as albatrosses, may have the capacity to migrate such vast distances while infected with H5N1. Moreover, some marine mammals, including Southern Elephant Seals and Leopard Seals, connect the Subantarctic islands with Oceania, as they migrate to the Southern coasts of Australia, including Tasmania, Victoria, and South Australia [67]. If these species can migrate vast distances while infected, the risk of viral transmission to mainland Australia remains a concerning possibility (Fig. 2).

## 6 Research gaps

### 6.1 Limited understanding of the emergence mechanisms driving the significant surge in HPAI outbreaks among Australian poultry in 2024, alongside a comprehensive historical overview of avian influenza in Australia

Although mainland Australia remains free from the dominant clade 2.3.4.4b HPAI H5N1, it has experienced continuous avian influenza outbreaks in poultry since 1976 [68]. Due to the relatively low number of occurrences compared to other regions [11], research on avian influenza in Australia remains limited. Simultaneously, surveillance efforts face considerable challenges, as targeted birds often reside in remote areas, exhibit unpredictable movement patterns, and have short infection durations that yield limited pathological data [69]. These constraints have led to an incomplete understanding of the broader avian influenza viral community in Australia.

However, the significant surge in HPAI outbreaks among poultry in Australia in 2024 has raised vigilance regarding the potential evolutionary mechanisms driving the shift [8,9]. Possible contributing factors may include changes in the primary flyways of wild birds, climate variability, or other ecological influences. Australia has long served as a major reservoir for LPAI, hosting various subtypes that have the potential to mutate into highly pathogenic strains through viral reassortment [70]. Moreover, the dynamics governing LPAI circulation are evolving. A recent study identified two distinct patterns in the newly introduced LPAI H4 and H10 viruses in Australia [71]. These shifting dynamics underscore the need for a holistic understanding of avian influenza in Australia. Focused research on examining historical occurrences is essential to reveal the mechanisms driving the recent surge in poultry outbreaks and to improve disease preparedness and response.

### 6.2 Lack of analyses of epidemiological determinants influencing avian influenza transmission within the unique Australian context

Most research on AIV ecology, molecular phylogenetics, and transmission dynamics comes from Asia, Europe, and North America, including studies on the dominant clade 2.3.4.4b HPAI H5N1 [67]. However, Australia's geographic isolation and distinct avian host ecology suggest that its epidemiology may differ from patterns observed in other regions. As a result, assumptions about AIV epidemiology based on overseas research may not be directly applicable to Australia [72].

Within the Australian context, waterfowl distributions are primarily driven by the availability of water bodies, particularly at interfaces where shorebirds and seabirds congregate [73]. As a result, key drivers of AIV emergence likely include rainfall patterns, wetland availability, and waterfowl distribution dynamics [74]. Australia's highly variable climate plays a significant role in shaping wetland availability, which may further influence waterfowl movement patterns [67]. Periods of drought may disrupt habitat connectivity, whereas flood events can expand congregation areas for waterbirds, increasing outbreak risks. Additionally, climate fluctuations, particularly influenced by the El Niño-Southern Oscillation and Southern Ocean currents, may alter habitat suitability, impacting migratory pathways and wetland availability, thereby shaping viral transmission dynamics [72].

### 6.3 Absence of integrated risk mapping for clade 2.3.4.4b HPAI H5N1 in Australian poultry, incorporating modelling approaches with migratory flyways and key epidemiological determinants

Mapping the risk of panzootic clade 2.3.4.4b HPAI H5N1 emergence in Australian poultry requires a comprehensive analysis of multiple contributing factors. Notably, the presence of HPAI outbreaks in Australia has largely been associated with three key elements: the adaptability of introducing virus strains to local hosts on arrival, the dispersal dynamics within Australia, and the distribution of poultry farms [11]. Consequently, risk mapping for Australian poultry necessitates a clear understanding of the relationships between these components, along with factors that may influence transmission. Previous studies have contributed valuable insights into the distribution of poultry farms in Australia, including observations that certain areas located in proximity to migratory flyways lack large-scale commercial farms [11]. However, evolving bird movement patterns, driven by dynamic epidemiological determinants, may lead to shifts in poultry outbreak dynamics. Thus, an integrated risk mapping framework for clade 2.3.4.4b HPAI H5N1 in Australian poultry is essential, incorporating key determinants to assess and anticipate transmission risks more accurately.

### 6.4 Lack of assessment of the pandemic risk associated with clade 2.3.4.4b HPAI H5N1

The World Health Organisation (WHO) Global Influenza Programme developed the Tool for Influenza Pandemic Risk Assessment (TIPRA) in 2016 to evaluate the viral characteristics of zoonotic influenza subtypes or subtypes with pandemic potential [74]. TIPRA is not designed to predict which influenza subtype will cause the next pandemic. Instead, its primary function is to evaluate a virus's evolving capability for human-to-human transmission. Since its update in 2020, TIPRA has been applied to various Influenza A subtypes, including H7N9, H9N2, and H5 clade 2.3.4.4 [75]. However, these tools have been used to a limited extent in assessing the pandemic risk posed by the dominant panzootic clade 2.3.4.4b HPAI H5N1 [74,75]. Given its remarkable mammalian adaptations, enabling infections across a wide range of hosts, and the rapid evolution of its epidemiological patterns in animal and human populations, the pandemic risk associated with clade 2.3.4.4b HPAI H5N1 is escalating. Consequently, a thorough risk assessment of this clade has become an urgent priority.

### 6.5 Limited evaluation of global disease burden of avian influenza & implications for One Health action

The global spread of the panzootic clade 2.3.4.4b HPAI H5N1 has led to extensive economic impacts. In particular, the dairy and poultry industries in the U.S. have experienced significant losses. In other countries, the impacts have extended beyond agriculture to affect trade, tourism, and food security services [76]. Although mainland Australia has not yet been affected by clade 2.3.4.4b H5N1, the resurgence and escalating frequency of HPAI H7 outbreaks among poultry have had notable consequences [9]. For instance, the recent detection of HPAI H7N8 at Kinross Farms- a major supplier of eggs to Australian supermarkets- resulted in the mass culling of birds as a control measure [77].

Despite these developments, the broader impacts of avian influenza remain inadequately analysed across economic, epidemiological, and ecological dimensions worldwide. Particularly, avian influenza, like clade 2.3.4.4b HPAI H5N1, poses a significant threat to Australia's unique wildlife, with the risk of causing the loss of endemic species. A comprehensive, multifaceted analysis of the disease burden may identify vulnerabilities and inform targeted One Health interventions. Given Australia's heavy reliance on international trade, its approach to addressing potential pandemic threats- particularly those posed by the dominant clade 2.3.4.4b HPAI H5N1- may require a shift from a historically reactive stance to a more proactive and forward-looking strategy. For example, when the WHO alerted to the pandemic potential of HPAI H5N1 in 2013, the Australian government allocated the majority of its investment to antiviral stockpiles and personal protective equipment [78]. In contrast, less than 10 % of funding was directed toward prevention and surveillance efforts in wild and domestic avian populations, where such viruses are most likely to emerge [78]. To strengthen the One Health approach, which emphasises the interconnectedness of human, animal, and environmental health, a more comprehensive and coordinated response is essential [78].

## 7 Conclusion

This review has established a foundational understanding of the clade 2.3.4.4b highly pathogenic avian influenza (HPAI) A H5N1 virus. It provided a comprehensive overview of the virus's emergence, the ongoing panzootic situation, and the latest findings on genomic analysis and pathogenesis in key animal models that govern its global spread. Additionally, the review examined epidemiological patterns in the affected regions, offering insights into the essential factors influencing the virus's potential incursion into mainland Australia. Such efforts lay the groundwork for further research aimed at risk mapping HPAI H5N1 clade 2.3.4.4b in mainland Australia, evaluating its pandemic potential, and informing One Health strategies for effective prevention and response.

## CRediT authorship contribution statement

**Pan Zhang:** Writing – original draft, Conceptualization. **C Raina MacIntyre:** Writing – review & editing, Supervision.

## Funding sources

PZ is supported by and acknowledges the Australian Government Research Training Program Scholarship and Balvi Filantropik fund. CRM is funded by NHMRC Investigator Grant 2016907.

## Declaration of competing interest

The authors declare that they have no known competing financial interests or personal relationships that could have appeared to influence the work reported in this paper.

## Data Availability

This study utlized data that are publicly available.
